# Supplementary material for: Dynamic transcriptomic profiles of zebrafish gills in response to zinc supplementation
Source: BMC Genomics. 2010 Oct 11;11:553. doi: 10.1186/1471-2164-11-553 (PMC3091702; doi:10.1186/1471-2164-11-553)
Supplement: Additional file 2 — Interactive Direct Interaction Network representing the molecular interactions between zinc, copper, iron, calcium and proteins encoded by transcripts changed by zinc supplementation. Mini web-site containing index.html and hyperlinked pages in subdirectory describing a Direct Interaction Network automatically generated based on curated interactions contained within the proprietary PathwayArchitect database. Ovals represent proteins and the circles symbolize metal ions. Objects are coloured by their abundance in zebrafish at the time-point they were significantly different from the control is a scale from -4 fold (dark green) to +4 fold (dark red). Where significant differences were found at more than one time-point, the colour overlay shows expression at the first instance. Dark blue squares denote 'binding', and light blue squares 'expression'; green squares stand for 'regulation', green diamonds for 'metabolism', and green circles for 'promoter binding'. Arrow heads indicate directionality of the interaction where annotated. All nodes and edges can be further interrogated by selecting the relative area of the image. [file 1471-2164-11-553-S2.zip › PathwayArchitect Zn xs DIN/403444.html]

# REGULATION:

|  |  |
| --- | --- |
| Type | REGULATION |
| Effect | Positive |


---

|  |  |
| --- | --- |
| Score | 0 |


---

|  |  |
| --- | --- |
| Reference Count | 9 |


---

|  |  |
| --- | --- |
| Mechanism | Unknown |


---

|  |  |
| --- | --- |
| Reference:0 || Sentence | "Ca2+-induced binding of the AP-1 factor to the regucalcin gene was completely inhibited by simultaneous administration of trifluoperazine, an antagonist of calmodulin, suggesting that the activation of nuclear AP-1 protein is partly mediated through a Ca2+/calmodulin-dependent pathway." |
| PMID | 9405289 |
| Year | 1998 |
| Species | Rat |
| Journal | Biochem J |
| RefScore | 1 |
| Source | PArchNLP |
  |
|  |  |


---

|  |  |
| --- | --- |
 Reference:1 || Sentence | "These results suggest that NMDA may potentiate AP1 binding following a sustained increase in intracellular free Ca(2+) concentrations through influxes across NMDA-operated and L-type voltage-sensitive Ca(2+) channels, in addition to release from intracellular Ca(2+) stores, in immature cultured rat hippocampal neurons." |
| PMID | 11835319 |
| Year | 2002 |
| Species | Rat |
| Journal | J Neurosci Res |
| RefScore | 1 |
| Source | PArchNLP |
  ||  |  |


---

|  |  |
| --- | --- |
 Reference:2 || Sentence | "These data show that [Mg2+]o may be an important, heretofore, overlooked natural modulator of proto-oncogene and NF-kappaB expression in VSMCs and that Ca2+ and PKC may play critical roles in induction of c-fos and c-jun in VSMCs induced by a decrease in [Mg2+]o." |
| PMID | 12944025 |
| Year | 2003 |
| Species | Rat |
| Journal | Am J Hypertens |
| RefScore | 3 |
| Source | PArchNLP |
  ||  |  |


---

|  |  |
| --- | --- |
 Reference:3 || Sentence | "The effect of L-glutamate (L-Glu) and its structural analogs N-methyl-D-aspartate (NMDA), quisqualate (QA), and kainate (KA) on the DNA binding activity of the Activator Protein 1 (AP-1) and the Ca2+/cAMP Responsive Element Binding Protein (CREB) families of transcription factors was examined in cultured chick retinal M�ller glia cells." |
| PMID | 7650753 |
| Year | 1995 |
| Journal | J Neurosci Res |
| RefScore | 3 |
| Source | PArchNLP |
  ||  |  |


---

|  |  |
| --- | --- |
 Reference:4 || Sentence | "We found that elevation of intracellular Ca2+ by ionomycin increased AP-1 DNA-binding activity in these cells." |
| PMID | 8954114 |
| Year | 1996 |
| Species | Mouse |
| Journal | Biochem Biophys Res Commun |
| RefScore | 0 |
| Source | PArchNLP |
  ||  |  |


---

|  |  |
| --- | --- |
 Reference:5 || Sentence | "Therefore, calcineurin negatively regulates Ca(2+)-stimulated AP-1 activity principally at the c-fos induction level." |
| PMID | 8954114 |
| Year | 1996 |
| Species | Mouse |
| Journal | Biochem Biophys Res Commun |
| RefScore | 0 |
| Source | PArchNLP |
  ||  |  |


---

|  |  |
| --- | --- |
 Reference:6 || Sentence | "The protein kinase inhibitor K252a abolished c-Jun induction and activator protein 1 (AP-1)-dependent reporter expression caused by Ca(2+) ionophore or hypoxia." |
| PMID | 11865053 |
| Year | 2002 |
| Species | Human |
|  | Mouse |
| Journal | Mol Cell Biol |
| RefScore | 0 |
| Source | PArchNLP |
  ||  |  |


---

|  |  |
| --- | --- |
 Reference:7 || Sentence | "In the present study, we determined whether Ca2+ signals and the activity of protein tyrosine kinases (PTKs) were required for the induction of c-jun in Jurkat cells stimulated with cross-linked anti-T cell receptor/CD3 antibodies or exposed to oxidative stress in the form of micromolar concentrations of H2O2." |
| PMID | 8640456 |
| Year | 1996 |
| Species | Human |
| Journal | J Interferon Cytokine Res |
| RefScore | 0 |
| Source | PArchNLP |
  ||  |  |


---

|  |  |
| --- | --- |
 Reference:8 || Sentence | In this study, we showed a biphasic effect of TG on osteoclast formation and apoptosis through the regulation of ROS production, caspase-3 activity, cytosolic Ca2+, and RANKL-induced activation of NF-kappaB and AP-1 activities. |
| Year | 2005 |
| PMID | 16007343 |
| Species | Mouse |
| Journal | J Bone Miner Res |
| RefScore | 2 |
| Source | PArchNLP |
  |


---

|  |  |
| --- | --- |
